# Prehospital ultrasound-guided nerve blocks improve reduction-feasibility of dislocated extremity injuries compared to systemic analgesia. A randomized controlled trial

**DOI:** 10.1371/journal.pone.0199776

**Published:** 2018-07-02

**Authors:** Benedikt Büttner, Ashham Mansur, Matthias Kalmbach, José Hinz, Thomas Volk, Karoly Szalai, Markus Roessler, Ingo Bergmann

**Affiliations:** 1 Department of Anesthesiology, Emergency and Intensive Care Medicine, University Medical Center, University of Goettingen, Goettingen, Germany; 2 Department of Anesthesiology and Intensive Care Medicine, Hospital of Fulda, University Medical Center of Marburg, Fulda, Germany; 3 Department of Anesthesiology, Intensive Care Medicine and Pain Medicine, University Medical Centre, Saarland University, Homburg (Saar), Germany; 4 Department of Trauma, Spine Surgery and Orthopedics, Evangelical Hospital Mülheim, Mülheim (an der Ruhr), Germany; Medizinische Universitat Graz, AUSTRIA

## Abstract

**Background:**

Out-of-hospital analgosedation in trauma patients is challenging for emergency physicians due to associated complications. We compared peripheral nerve block (PNB) with analgosedation (AS) as an analgetic approach for patients with isolated extremity injury, assuming that prehospital required medical interventions (e.g. reduction, splinting of dislocation injury) using PNB are less painful and more feasible compared to AS.

**Methods:**

Thirty patients (aged 18 or older) were randomized to receive either ultrasound-guided PNB (10 mL prilocaine 1%, 10 mL ropivacaine 0.2%) or analgosedation (midazolam combined with s-ketamine or with fentanyl). Reduction-feasibility was classified (easy, intermediate, impossible) and pain scores were assessed using numeric rating scales (NRS 0–10).

**Results:**

Eighteen patients were included in the PNB-group and twelve in the AS-group; 15 and 9 patients, respectively, suffered dislocation injury. In the PNB-group, reduction was more feasible (easy: 80.0%, impossible: 20.0%) compared to the AS-group (easy: 22.2%, intermediate: 22.2%, impossible: 55.6%; p = 0.01). During medical interventions, 5.6% [1/18] of the PNB-patients and 58.3% [7/12] of the AS-patients experienced pain (p<0.01). Recorded pain scores were significantly lower in the PNB-group during prehospital medical intervention (median[IQR] NRS PNB: 0[0–0]) compared to the AS-group (6[0–8]; p<0.001) as well as on first day post presentation (NRS PNB: 1[0–5], AS: 5[5–7]; p = 0.050). All patients of the PNB-group would recommend their analgesic technique (AS: 50.0%, p<0.01).

**Conclusions:**

Prehospital ultrasound-guided PNB is rapidly performed in extremity injuries with high success. Compared to the commonly used AS in trauma patients, PNB significantly reduces pain intensity and severity.

## Introduction

Joint luxation and dislocated fractures are frequently encountered in prehospital patient care and are often associated with extreme pain. Although pain is usually the most dominant presenting complaint in patients admitted to the emergency room, this symptom is commonly undertreated with “oligoanalgesia” especially in trauma victims [1]. An explanation for this restrictive approach to analgesic therapy even in patients with injuries that are not life-threatening is the avoidance of possible complications such as respiratory and hemodynamic changes arising from the use of anesthetics. Further, the use of these medications in such situations leads to increased requirements for monitoring and adequately trained medical personnel [1,2]. Certainly, an optimized pain management approach in trauma patients may lead not only to increased patient comfort but also has the potential to improve outcomes. Thus, enhanced focus on adequate analgesia, especially in trauma cases, has been shown to be associated with decreased morbidity [3,4]. Early fracture reduction, limiting complications and sequelae, may be the primary surgical objective. However, as reduction is extremely painful, sufficient analgesia and muscle relaxation are desirable to limit reduction attempts. In the vast majority of these cases, this is achieved through intravenous analgosedation (AS) [5]. Although, especially in the prehospital setting, emergency anesthesia is fraught with several major complications such as pulmonary aspiration and cardiovascular depression [6]. Peripheral nerve block (PNB) is a safe anesthetic technique that is increasingly being used in the operative management of extremity injuries and is proven to be beneficial when compared to systemic analgesia [7,8]. Despite the facts that PNB can be performed safely and rapidly and provides excellent pain relief and muscle relaxation, its use in the prehospital setting is extremely uncommon [1,2,9,10].

In this study, we examined the application and analgesic efficacy of ultrasound-guided PNB in prehospital trauma patients who present with injuries to the extremities. We hypothesize that in patients with an isolated injury of an extremity, the prehospital use of PNB will result in significantly lower pain scores on the day of trauma and the following two days, as well as improving the reduction-feasibility of dislocated injuries compared to that in patients who had received AS alone.

## Methods

This prospective study was approved by the ethics committee of the University Medical Center Goettingen (Amendment to No. 9/8/11) and was registered at the *Deutsches Register klinischer Studien* under the clinical trial number DRKS00009541 on February 09, 2016. The study was conducted within the Emergency Medical Services and the Helicopter Emergency Medical Services of Goettingen by emergency physicians (EP) of the Department of Anesthesiology of the University Medical Center Goettingen. The protocol for this trial (S1 File) and CONSORT checklist (S3 File) are available as supporting information.

### Patient recruitment

During a one-year period (February, 2016 to February, 2017), 30 prehospital trauma patients aged 18 years or older were included in the study. Inclusion criteria were an isolated injury of an extremity, causing pain (numeric rating scale NRS>3, see below) and requiring a prehospital medical intervention (e.g. reduction, splinting, technical rescue). Patients had to be able to give informed consent. Exclusion criteria were any preexisting nerve damages of the extremities or known allergy to local anesthetics. At the scene of the accident, verbal informed consent for participation in the study was obtained. A written consent was later obtained during the course of hospital stay. Prehospital treatment of patients was managed by two EPs (IB, MK), who work also as consultant anesthetists and are certified by the *German Society of Anesthesia and Intensive Care* (DGAI) to practice ultrasound-guided regional anesthesia. At the point of first contact, a structured physical examination was performed for all patients. Standard clinical monitoring was established (non-invasive blood pressure, pulse oximetry, continuous electrocardiography) and peripheral venous access was ensured. By the toss of a coin at the scene of accident, patients were randomly assigned to the study group (PNB) to have a PNB proximal to injury location or to the control group (AS) for AS alone. Motor function, vascularisation and sensitivity of the injured extremity were examined before analgesic procedure and after the required medical intervention at the scene of accident.

### Intravenous analgosedation (AS)

Intravenous analgesia was usually performed by the attending EP using a standard technique of s-ketamine (0.125–0.25 mg /kg body weight, BW) combined with midazolam (0.05 mg/kg BW). In some cases, fentanyl was combined with midazolam administered by the previous acting EP. In the event of additional demand for the trial EP of Helicopter Emergency Medical Services by this previous acting EP, the doses were adjusted to AS already administered (s-ketamine 0.125–0.25 mg /kg BW, fentanyl 0.5–1.5 μg/kg BW, midazolam 0.05 mg/kg BW; total dosages) to avoid mixture of several narcotic drugs and subsequent adverse events.

### Single shot peripheral nerve block (PNB)

A combination of femoral nerve block (2–3 cm distal to the inguinal ligament, short axis, out-of-plane) and sciatic nerve block (mid femoral lateral approach, short axis, in-plane) was performed for below-knee and foot injuries. Injuries of the thigh or patella were treated with a femoral nerve block. An interscalene brachial plexus block (superior and middle trunk, short axis, out-of-plane) was performed for the injuries of the upper extremity involving the shoulder and upper arm. Forearm injuries were treated with infraclavicular brachial plexus block (all fascicles, short axis, out-of-plane). Therefore, the respective targeted nerve/trunks/fascicles were located by ultrasound (12 MHz transducer, M-Turbo, FUJIFILM SonoSite, Bothell, USA). To ensure safe aseptic conditions, the PNB-procedures were conducted according to standard operating procedures that are comparable to those used in emergency departments. The puncture site was disinfected and anesthetized with 1% mepivacaine. The EP used steril gloves. Under ultrasound guidance, a 19 G stimulating cannula (55 mm, B. Braun Melsungen AG, Melsungen, Germany) was directed to the respective nerve/trunks/fascicles. After confirmation of correct needle tip position by ultrasound, 10 mL of prilocaine (1%) and 10 mL of ropivacaine (0.2%) were injected (single injection technique) until the entire circumference of the nerve/trunks/fascicles was shown to be surrounded by the local anesthetic [11].

### Data assessment

Vital parameters were regularly recorded. Patient characteristics including location (upper/lower limb) and type of injury (dislocation/closed fracture/open fracture) were documented as well as intensity of pain at arrival of the trial EP. Pain scores were recorded using a numerical rating scale (NRS 0 = no pain to 10 = worst pain imaginable) on first contact with the trauma patient by the trial EP. Intravenously administered analgesics were documented. After induction of the respective analgesic procedure, onset of action of PNB (time from infiltration of local anesthetic to loss of perception to cold stimulus in relevant area of innervation and NRS<3) or AS (time from intravenous administration of analgesics to their onset of action evaluated clinically by mobilizing the limb and paying attention to patient’s reaction) was recorded. In cases in which reduction was required, technical performance of the maneuver was classified by the managing trial EP as *easy*, *intermediate*, or *impossible*. On the second day post-intervention, the patients were interviewed retrospectively by a blinded study doctor. Herein, patients were asked, if they felt any pain at all at the scene of accident, immediately after specific analgesic procedure, during the prehospital medical intervention as well as at rest on the day of accident, on the first and on the second day after the accident. In those patients with pain the average pain intensity was recorded for given events and time points. In the ward of the trauma surgery departement of the University Medical Center of Goettingen patients were given ibuprofen (600 mg p.o., three times a day) according to standardized protocol. Oxycodone/naloxone (10 mg/5 mg p.o., twice daily) was prescribed if further analgesia was necessary.

Symptoms and signs of nerve damage of the affected extremity were elicited. Patient satisfaction with specific analgesic procedure (Likert scale of 1 = very satisfied to 6 = very unsatisfied) was recorded and the patients were asked, if they would recommend the analgesic technique.

### Statistical analysis

The primary outcome was presence of pain [yes/no] during the prehospital medical intervention. Secondary outcomes were feasibility of reduction and associated pain scores during prehospital medical intervention, at the scene of accident, immediately after specific analgesic procedure and at rest on the whole day of accident, on the first and on the second day after the accident. Further secondary outcomes were complications (e.g. nerve damage, compartment syndrome) and patient satisfaction with the specific analgesic procedure. Based on prior observations, a power analysis revealed that 12 patients in each group would be sufficient to permit in 95% a painless prehospital intervention in the PNB-group compared to 45% in the AS-group (power: 0.8, significance level: 0.05). The data were analyzed with the statistics program StatSoft® (Dell Inc., Texas, USA). Continuous data were tested for normal distribution with the Kolmogorov-Smirnov test. Normally distributed data were described with mean and standard deviation, and others with median and interquartile range (IQR). Categorical data were given as percentages. Normally distributed data were compared with the two-tailed Student t-test for independent samples, and non-normal data with Mann-Whitney U-test. Categorical data were compared with two-tailed Fisher's exact test and with two-tailed Pearson's Chi-square test. A p-value <0.05 was defined as statistically significant.

## Results

**Fig 1:**
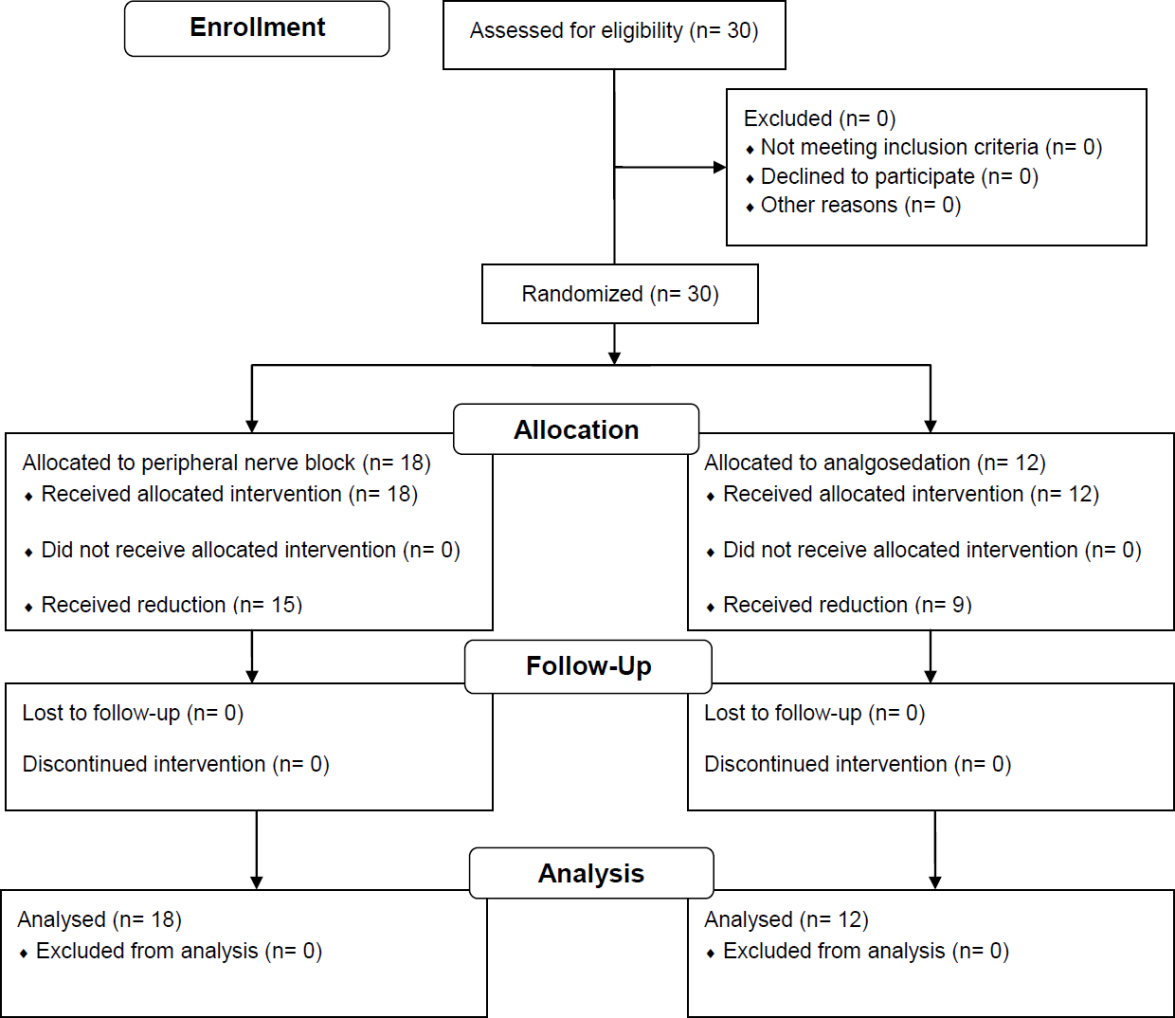
Flow diagram summarizing the study design.

Thirty patients were screened for eligibility and were randomized. All 30 prehospital emergency patients were included in the study. Eighteen patients were allocated to the study group (PNB) and 12 patients were allocated to the control group (AS) (Fig 1).

Both groups exhibited similar anthropometric data and no significant difference was noted with respect to the American Society of Anesthesiology (ASA) classification (Table 1).

**Table 1:** Patient characteristics.

	PNB	AS	
	n = 18	n = 12	p
Age (years) (means±SD)	53.3±17.3	51.9±22.0	0.85
Gender (male/female), n	13/5	6/6	0.27*
Height (cm) (means±SD)	176.6±6.0	175.0±10.1	0.58
Weight (kg) (means±SD)	87.4±10.9	80.9±14.2	0.17
ASA (I/II/III), n	12/3/3	8/4/0	0.23^#^

PNB: peripheral nerve block; AS: analgosedation; SD: standard deviation; ASA: American Society of Anesthesiology

* two-tailed Fisher’s exact test

^#^ two-tailed Pearson's Chi-square test

Vital parameters such as heart rate and peripheral saturation, which were documented before the analgesic intervention, were found to be evenly distributed. A difference was noted in the initially recorded blood pressure (Table 2). Furthermore, patients of the PNB-group tended to have lower heart rate, which is indicative for positive hemodynamic side effects (see S1 Table).

**Table 2:** Heart rate, blood pressure, oxygen saturation (means±SD)^$^.

	PNB	AS	
	n = 18	n = 12	p
Initial heart rate (min^-1^)	98.6±17.0	91.7±13.8	0.24
Initial systolic blood pressure* (mmHg)	133.8±29.9	160.7±26.8	0.02
Initial diastolic blood pressure* (mmHg)	77.6±9.0	90.2±10.3	<0.01
Initial peripheral oxygen saturation (%)	96.9±3.2	97.7±1.4	0.40
Heart rate post analgesic procedure* (min^-1^)	78.6±11.0	87.7±31.8	0.28
Systolic blood pressure post analgesic procedure* (mmHg)	139.4±15.9	142.1±22.6	0.71
Diastolic blood pressure post analgesic procedure* (mmHg)	81.5±10.3	90.8±28.5	0.22
Peripheral oxygen saturation post analgesic procedure (%)	98.6±0.5	95.2±5.2	<0.01

PNB: peripheral nerve block; AS: analgosedation; SD: standard deviation

^$^ two-tailed Student t-test

* data of one patient missing

After the analgesic procedure, no significant difference was observed in the hemodynamic parameters (Table 2). However, a significantly lower peripheral saturation was recorded in the control group (mean±SD, PNB: 98.6±0.5%, AS: 95.2±5.2%; p<0.01). Pronounced hypoxemia (SpO_2_<90%) occurred in two AS-patients, but saturation quickly increased after increasing the oxygen flow rate.

The following PNBs were used in patients with upper extremity injuries: interscalene brachial plexus block (n = 1), infraclavicular brachial plexus block (n = 1). The distribution of the PNBs in patients with lower extremity injuries were: combination of femoral nerve block and sciatic nerve block (n = 12), single femoral nerve block (n = 4). In the PNB-group systemic analgesia was already administered by a previous acting EP in 16 patients (88.9%), while the trial EP was additionally demanded. Table 3 summarizes the injury patterns in both groups.

**Table 3:** Injury patterns and feasibility of the reduction maneuver.

	PNB	AS	
	n = 18	n = 12	p
Upper extremity, n (%)	2/18 (11.1)	4/12 (33.3)	0.18*
Lower extremity, n (%)	16/18 (88.9)	8/12 (66.7)	0.18*
thigh, n (%)	4/18 (22.2)	3/12 (25.0)	1.00*
lower thigh, n (%)	12/18 (66.7)	5/12 (41.7)	0.26*
Dislocated injury and reduction required, n (%)	15/18 (83.3)	9/12 (75.0)	0.66*
Reduction-feasibility	n = 15	n = 9	
easy, n (%)	12/15 (80.0)	2/9 (22.2)	0.01*
intermediate, n (%)	0/15 (0.0)	2/9 (22.2)	0.13*
impossible, n (%)	3/15 (20.0)	5/9 (55.6)	0.10*
			0.01^#^

PNB: peripheral nerve block; AS: analgosedation

* two-tailed Fisher’s exact test

^#^ two-tailed Pearson's Chi-square test

Twenty-four patients presented with dislocation injuries; prehospital reduction was performed under PNB in 15 patients (83.3%) and under AS in 9 patients (75.0%; p = 0.66). No difference was seen in the onset of action of the analgesic technique between the two groups (means±SD; PNB: 2.7±0.9 min, AS: 2.2±0.9 min; p = 0.18). The feasibility of prehospital reduction maneuver was described as *easy* by the trial EP in 80.0% [12/15] of the PNB-patients and as *impossible* in the remaining 20.0% [3/15] of patients. On the contrary, only 22.2% [2/9] of reduction maneuvers were described as *easy*, and another 22.2% [2/9] as *intermediate*, while 55.6% [5/9] of maneuvers were assessed as *impossible* (p = 0.01).

During this prehospital medical intervention, only 5.6% of the PNB-patients [1/18] in contrast to 58.3% of the AS-patients [7/12] experienced pain (p<0.01), and the recorded pain score was less in the PNB-group (median[IQR] NRS PNB: 0[0–0], AS: 6[0–8]; p<0.001). Patients from both groups reported severe pain at the scene of accident before the analgesic procedure (Table 4). Immediately afterwards pain intensity was lower in the PNB-group (median[IQR] NRS 0[0–3]) as compared to the control group (AS: NRS 0.5[0–7.5]; p<0,01), and 5 of 12 patients of the AS-group presented a NRS>5. Recorded pain intensity was significantly lower in the PNB-patients compared to the AS-group the prehospital medical intervention as well as during clinical diagnostic procedures post hospital admission (Table 4). Pain scores at rest on the day of accident and on first day post-accident were also lesser in the PNB-group, while there was no significant difference on the second day post presentation regarding the two groups (Table 4).

**Table 4:** Pain intensity and patient satisfaction.

	PNB	AS	
	n = 18	n = 12	p
Initial pain score (NRS 0–10)	8 [7–9]	9.5 [9–10]	0.14^§^
Pain post analgesic procedure (NRS 0–10)	0 [0–3]	0.5 [0–7.5]	<0.01^§^
Patients with pain during medical intervention at the site of accident, n (%)	1/18 (5.6)	7/12 (58.3)	<0.01*
Pain during medical intervention at the site of accident (NRS 0–10)	0 [0–0]	6 [0–8]	<0.001^§^
Reduction required	n = 15	n = 9	
Patients with pain during reduction, n (%)	1/15 (6.7%)	3/9 (33.3%)	0.13*
Pain (if present) during reduction (NRS 1–10)	3 [3–3]	10 [8–10]	0.04^§^
Pain during clinical diagnostics (NRS 0–10)	0 [0–0]	5 [1.5–8]	<0.001^§^
Patients with pain on day of accident, n (%)	4/18 (22.2)	9/12 (75.0)	<0.01*
Pain on day of accident (NRS 0–10)	0 [0–0]	7.5 [2.5–8]	<0.001^§^
Patients with pain on first day post-accident, n (%)	9/18 (50.0)	11/12 (91.7)	0.02*
Pain on first day post-accident (NRS 0–10)	1 [0–5]	5 [5–7]	0.050§
Patients with pain on second day post-accident, n (%)	10/18 (55.6)	8/12 (66.7)	0.71*
Pain on second day post-accident (NRS 0–10)	2 [0–5]	4.5 [0–5]	0.41^§^
Satisfaction (Likert scale 1–6)	1.2±0.6	2.0±1.5	0.06^§^
Patients who would recommend their technique, n (%)	18/18 (100)	6/12 (50.0)	<0.01*

mean±SD; median [IQR]; PNB: peripheral nerve block; AS: analgosedation; SD: standard deviation; NRS: numeric rating scale

* two-tailed Fisher’s exact test

^§^ Mann-Whitney U test

In both groups patients exhibited no signs of persisting neurological deficits or post-intervention compartment syndrome in the affected extremity. Patients’ satisfaction with regard to the analgesic technique selected was (means±SD) 1.2±0.6 in the PNB-group and 2.0±1.5 in the AS-group (p = 0.06). All patients of the PNB-group and only 50.0% of the AS-group would recommend their respective technique (p<0.01).

## Discussion

In this study, we compared the effects of prehospitally administered PNB to AS alone in trauma patients who presented with an isolated extremity injury. We investigated pain intensity during and up to two days after an emergency procedure at the scene of accident, and the effects of the respective analgesic approach on the feasibility of a prehospital reduction of dislocation injuries of the extremities. Our results suggest that ultrasound-guided PNB can be used expediently in the prehospital emergency management of extremity injuries. Patients who received a PNB not only experienced less pain but the necessary reduction procedures were more easily performed than in patients who received AS alone.

Pain is one of the leading complaints of trauma patients [4]. Nevertheless, in the prehospital settings and in emergency departments, pain is often inadequately treated in these patients, with the intention of avoiding complications associated with commonly used opioid therapy [1,2]. Peripheral nerve blockade is a well-established approach for intra- and post-operative pain management in the surgical treatment of extremity injuries [8]. With regard to the adverse events and patient safety, PNBs are superior to systemic anesthetic procedures due to their minimal complication rate as well as their excellent respiratory and hemodynamic stability in the management of emergency patients [1,7]. While there were no adverse effects in any patient, our study was not powered to look at safety.

PNB has long been performed with success in the military trauma patients [10]. Despite the fact that recent literature has worked out the advantageous and practical use of femoral PNB indicated in patients with certain leg injuries in comparison with AS, this nerve block is seldom performed in the emergency department [12–14]. Even though, in current literature the more frequent use of PNBs outside the operating theatre is required [1,2,9,10], regional anesthetic techniques are used occasionally in the prehospital setting [12,15–22]. Sufficient pain management is essential to be able to perform necessary prehospital interventions (e.g. reduction). However, these situations present their own inherent and often new challenges to the medical personnel. In general, the prehospital analgesic approach is conducted with systemic analgesia whereby serious entailed complications have to be expected and their management has to be mastered [6]. These complications could almost completely be avoided by the use of PNBs. Our results as well as the data from previous out-of-hospital studies suggest that PNBs independent of patient age can provide satisfactory, complication free pain management directly in the prehospital setting [12,17,18,20,21]. No patient in either the AS- or the PNB-group experienced an adverse hemodynamic event after the induction of the respective analgesic procedure. However, significantly reduced oxygen saturations in the control group (AS) were detected and two of these patients experienced profound but easily managed oxygen desaturations. In addition to our study, only McRae et al. [21] previously compared AS (morphine use) and PNB (fascia iliaca compartment block) in the prehospital setting. Both studies show that the patients who already received a PNB at the scene of accident experienced significantly reduced pain.

McRae and co-workers [21] compared prehospitally administered PNB with AS solely in patients with femoral fractures. Our results indicate first that PNB is superior to AS as a form of pain control in almost all patterns of injuries to the extremities. Furthermore, PNB-patients not only had a lower incidence of pain but also less severe pain during required medical intervention (including reduction of dislocated injuries) at the site of accident and up to the day one post intervention. Indeed, most patients from the PNB-group also received systemic analgesia; thus, an additive effect from both procedures may be assumed. Nevertheless, directly after the hospital admission, a marked difference in pain could be observed between the two groups. Those patients who had received AS complained of pain much earlier and at higher intensities. We have therefore excluded a relevant summative effect due to the combination of PNB and AS. Such persisting analgesia after PNB has recently been shown after ambulatory arthroscopies [23]. Büttner and co-workers [23] suggest PNBs prevent nociceptive input following central sensitization. A similar mechanism of action is also thought to occur here, since AS-patients experienced more frequent and more intense pain despite the AS being performed by trained EPs in anesthesia, where AS lies within their daily capability. Therefore, an almost adequate analgesia can be expected at best.

Successful reduction requires adequate analgesia and muscle relaxation. With regard to the outcome of the dislocated extremity injuries, it is especially interesting how much relief a PNB affords the undertaking of a necessary reduction. Only two previous studies have compared the success rate of shoulder reduction in the emergency department following PNB or AS, and reported equivalent good results [24,25]. Both studies showed high success rates for shoulder reduction after interscalene brachial plexus block and after a suprascapular nerve block. Despite smaller patient numbers, our study shows significantly more feasible repositioning of dislocated fractures or joints after a PNB in comparison with AS. This difference from previous studies in the emergency department [24,25] may possibly be due to the prehospital setting in our study in which the generally difficult conditions more pronounce the advantages of a PNB. Underhill et al. [26] have also demonstrated that 87% of the patients with shoulder subluxation can be repositioned under just interscalene brachial plexus block and without additional administration of intravenous analgesic drugs. However, complete painlessness was observed only in 47% of the studied patients [26]. The reason why we were able to achieve better results with regard to pain control may rely on the fact that in previous studies, the landmark-based technique was used for PNB. The vast majority of available studies investigating PNB conducted in prehospital settings or emergency departments have used the landmark-based technique for femoral nerve block, whereas conducting PNB of the upper extremity in these settings was very rare [1,9]. To our knowledge, this is the first randomized trial performing PNB in a prehospital setting with the ultrasound-guidance technique. Dochez et al. [12] have already speculated on benefits of the ultrasound-guided technique in prehospital settings. Consecutively, carrying out prehospital PNB can be suggested to treat patients with upper extremity fractures as hitherto shown only in a case report [16]. The fact that the anesthesiologists who were involved in conducting this study were very experienced and had additional qualifications in ultrasound-guided regional anesthesia should not discourage others who are not that familiar with this technique to start establishing it. That is because feasibility studies from the emergency department showed that the ultrasound-guided PNB is easy to learn [27].

There are still major concerns among clinicians with regard to PNB in patients with extremity fractures explained by their worries that PNP may mask the early symptom of compartment syndrome “pain” and miss timely diagnosis [1,2,9,10]. In contrast to this, there is a misleading focus on pain or paresthesia for diagnosing a compartment syndrome because of their bad usability on the one hand. Furthermore, through painlessness reached by PNB new ischemic breakthrough pain triggered by compartment syndrome, it could be detected much easier. Thus, PNB could provide even benefits as an additive procedure to compartmental-pressure monitoring to diagnose compartment syndrome [1,9,10].

There are some limitations of this study. In addition to the relatively small trial, the number of subjects in the samples were not equivalent. There were different types of medical interventions required for the patients of each group. Due to already administered AS by primary acting EPs a standardized intravenous AS could not be performed. The nerve block procedure may trigger a placebo-effect in the study group, which only can be excluded by perineural injection of saline solution in the control group. Because of aggravated circumstances in the prehospital settings the PNB-performing EPs have to exhibit a high education level in ultrasound-guided regional anesthesia. Furthermore, prehospital care in some countries (e.g. the United States of America) is provided by paramedics, who are unlikely to acquire the expertise required for ultrasound-guided PNB in the field.

In conclusion, this study demonstrates for the first time that prehospital ultrasound-guided PNB can be conducted with high success. PNBs could be extended to almost all patterns of injuries to the extremities. In addition to several investigations on performance of PNB in the emergency departments or prehospital settings, we could show that PNBs significantly reduce pain intensity and severity in trauma patients. Particularly, by using ultrasound-guided PNB, prehospital reduction-feasibility of dislocated extremity injuries, and presumably the success rate of reduction, can be improved.

## Supporting information

S1 File English translation of the study protocol. (PDF)

S2 File Original study protocol in German language. (PDF)

S3 File CONSORT checklist of the study. (DOC)

S1 Table Trial data. (XLSX)
