# Supplementary material for: Prehospital ultrasound-guided nerve blocks improve reduction-feasibility of dislocated extremity injuries compared to systemic analgesia. A randomized controlled trial
Source: PLoS One. 2018 Jul 2;13(7):e0199776. doi: 10.1371/journal.pone.0199776 (PMC6028078; doi:10.1371/journal.pone.0199776)
Supplement: S1 File — (PDF) [file pone.0199776.s001.pdf]

Date:

Study protocoll: Prehospital peripheral nerve blocks (PNB)

Patient data: **Phone:**

Initials:	Sex:	Age:
ASA:	Weight:	Height:
Trauma:	Open / closed fracture:<br><br>Dislocated injury and reduction required (yes/no):	Pain according to NRS (1-10) before analgesic approach:<br><br>Reposition (easy / intermediate / impossible):

Group 1: regional anesthesia, PNB

Puncture site:		Local anesthetic:	
Time till onset of action:		Effect A/B/C:	
Sensitivity		Dose of s-ketamine / fentanyl (by previous acting EP):	
Motor function		Dose of midazolam (by previous acting EP):	

Group 2: analgosedation:

Drug doses were documented in the medical record of the trial emergency physician

<u>Both groups:</u>

Process times according to the medical record of the trial emergency physician

Vital parameter:

	initial	post analgesic approach	minimal	maximal
Heart rate:				
Blood pressure:				
SpO2:				

Interview, on second day post-intervention

1. Pain during during the prehospital medical intervention

1.1 Did you feel pain during the prehospital medical intervention? **Yes** ○ **No** ○

1.1.1 If so, please indicate pain severity? *(Select pain severity, whereat value ‚0' corresponds to no pain and value ‚10' corresponds to means worst pain imaginable)*

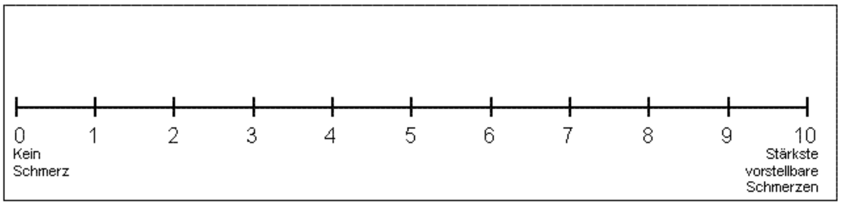

2. Pain post-intervention

2.1 Have you taken analgesics after the prehospital medical intervention? **Yes** ○ **No** ○

2.1.1 If so, which analgesics have you taken at which day post-intervention?

- Ibuprofen **Day of accident** ○ **1. day post-intervention** ○ **2. day post-intervention** ○

Which daily intake? **Day of accident** ___ **1. day post-intervention** ___ **2. day post-intervention** ___

- Oxycodone/naloxone **Day of accident** ○ **1. day post-intervention** ○ **2. day post-intervention** ○

Which daily intake? **Day of accident** ___ **1. day post-intervention** ___ **2. day post-intervention** ___

- Other **Day of accident** ○ **1. day post-intervention** ○ **2. day post-intervention** ○

Which daily intake? **Day of accident** ___ **1. day post-intervention** ___ **2. day post-intervention** ___

These questions relate pain after prehospital medical intervention:

3.1 Did you have pain **immediatly after the prehospital medical intervention**?

Yes ○ **No** ○

3.1.1 If so, please indicate pain severity? *(Select pain severity, whereat value ‚0' corresponds to no pain and value ‚10' corresponds to means worst pain imaginable)*

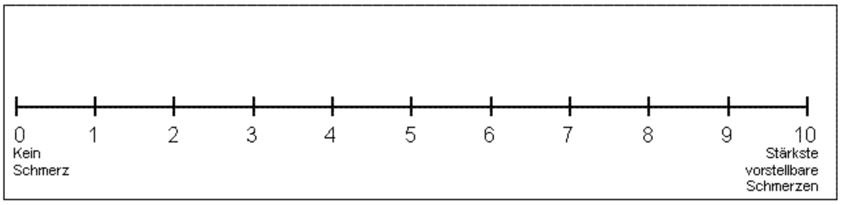

3.2 Did you have pain **on first day post-intervention**?

Yes ○ **No** ○

3.2.1 If so, please indicate pain severity? *(Select pain severity, whereat value ‚0' corresponds to no pain and value ‚10' corresponds to means worst pain imaginable)*

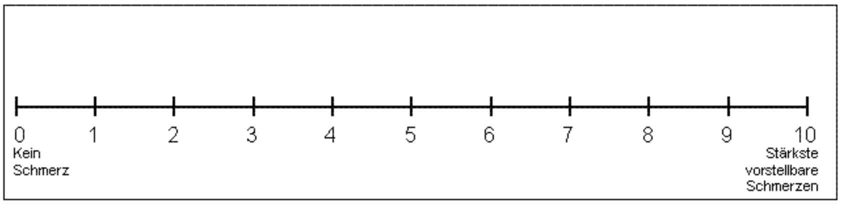

3.3 Did you have pain **on second day post-intervention**?

Yes ○ **No** ○

3.3.1 If so, please indicate pain severity? *(Select pain severity, whereat value ‚0' corresponds to no pain and value ‚10' corresponds to means worst pain imaginable)*

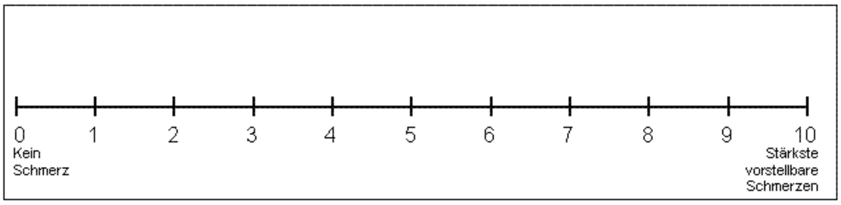

3.4 Did you have pain **during the prehospital medical intervention**?

Yes ○ **No** ○

3.4.1 If so, please indicate pain severity? *(Select pain severity, whereat value ‚0' corresponds to no pain and value ‚10' corresponds to means worst pain imaginable)*

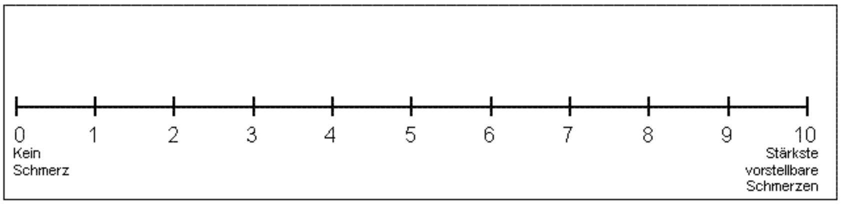

These questions relate complications after the prehospital medical intervention:

3.5 Did you experience any discomfort like tingling, deafness or paralysis of the injured limb from the first day after prehospital medical intervention?

Yes ○ **No** ○

3.5.1 If so, how long did this discomfort last? ___________________

3.5.2 If so, which specific discomfort did you experience? ___________________

4. Satisfaction with the specific analgesic procedure

4.1 Were you satisfied with your specific analgesic procedure during the prehospital medical intervention?

(Likert scale of 1=very satisfied to 6=very unsatisfied)

Likert scale (1 - 6): __

4.2 Would you recommend your specific analgesic procedure? **Yes** ○ **No** ○

Thank you very much for your support!

Your responsible emergency physician
