# Supplementary material for: Prehospital ultrasound-guided nerve blocks improve reduction-feasibility of dislocated extremity injuries compared to systemic analgesia. A randomized controlled trial
Source: PLoS One. 2018 Jul 2;13(7):e0199776. doi: 10.1371/journal.pone.0199776 (PMC6028078; doi:10.1371/journal.pone.0199776)
Supplement: S2 File — (PDF) [file pone.0199776.s002.pdf]

Datum:

Studienprotokoll: Ultraschall Blockaden Notfallmedizin

Patientendaten: **Tel.Nr.:**

Initialen:	Geschlecht:	Alter:
ASA:	Körpergewicht:	Körpergroße:
Trauma:	Offen / geschlossen<br><br>Disloziert (ja/nein)	NRS Vor Analgesie:<br><br>Reposition (Gut/mäßig/Nicht möglich)

Gruppe 1: Regionalanästhesie

Nadelgröße:		Punktionsversuche:	
Punktionsort:		Menge/Konz LA:	
NRS nach Block:		Parästhesien:	
Anlagezeit:		Effekt:	
Sens. Testung		Dosis Analgesie:	
Motor.Testung		Dosis Dormicum:	

Beide Gruppen:

Prozesszeiten:

Alarm:		Übergabe NA:	
Ankunft EST:		Einsatzbereit:	
Beginn Analgesie:		Beginn Diagnostik:	
Wirkung Analgesie:		Beginn OP / Versorgung:	
Abfahrt EST:		Beginn ANÄ:	

Ankunft KH:		Freigabe ANÄ:	

Parameter:

	Ausgangswert	Nach Analgesie	Min	Max
HF:				
RR:				
SpO2:				

Komplikationen / Klinik

	Analogskale zwischen 1-10
Vasopressor (Akrinor) ml (1-10)	
Menge Infusionsvolumen (ml):	
Kraftgrad 5 / Stunden:	
Größter Schmerz in NA / Diagnostik:	

Fragebogen, 2 Tage postoperativ

1. Vor dem Eingriff

1.1 Hatten Sie vor dem Ereigniss Schmerzen? **Ja** ○ **Nein** ○

1.1.1 Wenn ja wo? ______________________

1.1.2 Wie stark würden Sie die Schmerzen beschreiben? *(Markieren Sie die Stärke des Schmerzes, wobei der Wert ‚0' kein Schmerz und ‚10' der stärkste vorstellbare Schmerz sei)*

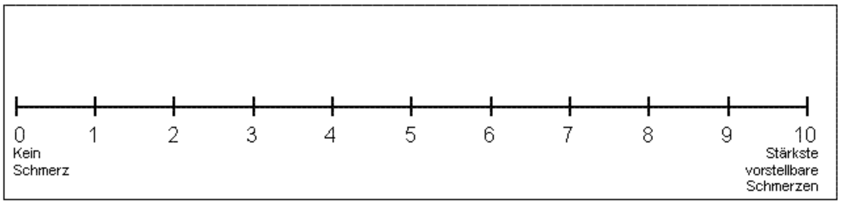

1.2 Haben Sie vor dem Ereigniss regelmäßig Schmerzmittel eingenommen? **Ja** ○ **Nein** ○

1.2.1 Wenn ja, welche? ______________________________

1.2.2 Wie lange haben Sie die Schmerzmedikamente eingenommen? __________________

1.3 Haben Sie vor diesem Ereigniss Vollnarkosen gehabt? **Ja** ○ **Nein** ○

1.3.1 Wenn ja, gab es Komplikationen mit der Vollnarkose?____________________________

1.4 Haben Sie vor diesem Ereigniss eine Regionale Betäubung gehabt? **Ja** ○ **Nein** ○

1.4.1 Wenn ja, gab es Komplikationen mit der Regionalanästhesie?______________________

2. Während des Eingriffes

2.1 Haben Sie während des Ereignisses Schmerzen verspürt? **Ja** ○ **Nein** ○

2.1.1 Wenn ja, wie stark waren diese Schmerzen? *(Markieren Sie die Stärke des Schmerzes, wobei der Wert ‚0' kein Schmerz und ‚10' der stärkste vorstellbare Schmerz sei)*

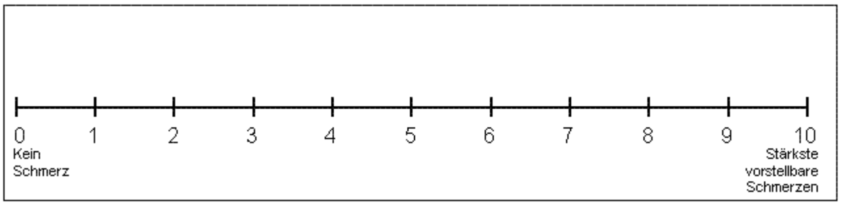

3. Nach der Intervention

3.1 Haben Sie in den ersten Drei Tagen nach der Intervention Schmerzmittel eingenommen?

Ja ○ **Nein** ○

3.1.1 Wenn ja, welche Schmerzmittel haben Sie an welchem Tag eingenommen?

- Diclofenac oder Ibuprofen **OP Tag** ○ **1. Tag post OP** ○ 2. Tag post OP ○

In welcher Tagesdosierung?: **OP Tag** _____ **1. Tag post OP** _____2. Tag post OP ______

- Novalmin **OP Tag** ○ **1. Tag post OP** ○ 2. Tag post OP ○

In welcher Tagesdosierung?: **OP Tag** _____ **1. Tag post OP** _____2. Tag post OP ______

- Targin **OP Tag** ○ **1. Tag post OP** ○ 2. Tag post OP ○

In welcher Tagesdosierung?: **OP Tag** _____ **1. Tag post OP** _____2. Tag post OP ______

- Tramal **OP Tag** ○ **1. Tag post OP** ○ 2. Tag post OP ○

In welcher Tagesdosierung?: **OP Tag** _____ **1. Tag post OP** _____2. Tag post OP ______

- Andere:_____________ **OP Tag** ○ **1. Tag post OP** ○ 2. Tag post OP ○

In welcher Tagesdosierung?: **OP Tag** _____ **1. Tag post OP** _____2. Tag post OP ______

Diese Fragen betreffen Schmerzen nach dem Eingriff:

3.3 Haben Sie **direkt nach der Intervention** oder **noch am selben Tag**

Schmerzen verspürt? **Ja** ○ **Nein** ○

3.3.1 Wenn ja, wie stark waren diese Schmerzen? *(Markieren Sie die Stärke des Schmerzes, wobei der Wert ‚0' kein Schmerz und ‚10' der stärkste vorstellbare Schmerz sei)*

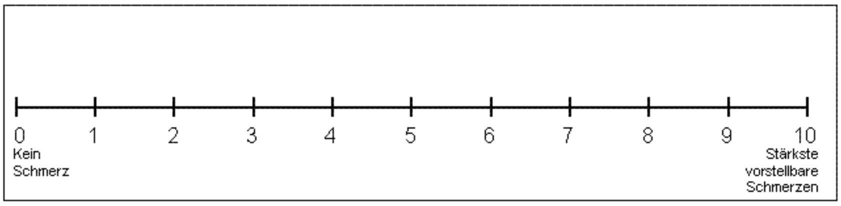

3.4 Haben Sie **am 1. Tag nach der Intervention** Schmerzen verspürt? **Ja** ○ **Nein** ○

3.4.1 Wenn ja, wie stark waren diese Schmerzen? *(Markieren Sie die Stärke des Schmerzes, wobei der Wert ‚0' kein Schmerz und ‚10' der stärkste vorstellbare Schmerz sei)*

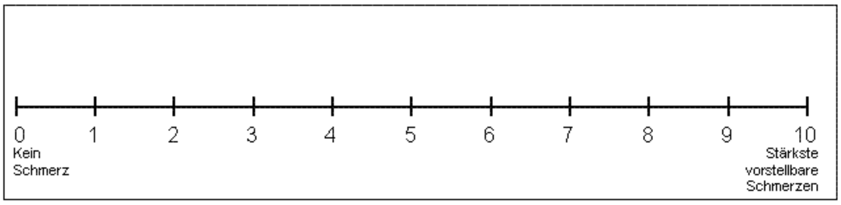

3.5 Haben Sie **am 2. Tag nach der Intervention** Schmerzen verspürt? **Ja** ○ **Nein** ○

3.5.1 Wenn ja, wie stark waren diese Schmerzen? *(Markieren Sie die Stärke des Schmerzes, wobei der Wert ‚0' kein Schmerz und ‚10' der stärkste vorstellbare Schmerz sei)*

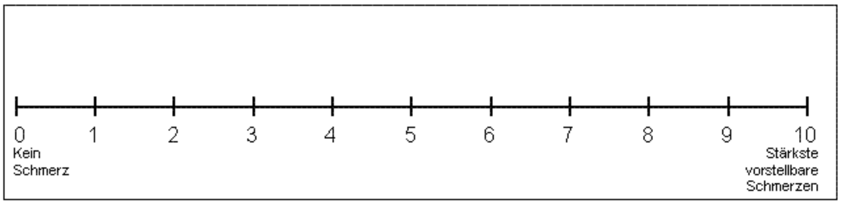

3.6 Haben Sie **während der Reposition** Schmerzen verspürt? **Ja** ○ **Nein** ○

3.5.1 Wenn ja, wie stark waren diese Schmerzen? *(Markieren Sie die Stärke des Schmerzes, wobei der Wert ‚0' kein Schmerz und ‚10' der stärkste vorstellbare Schmerz sei)*

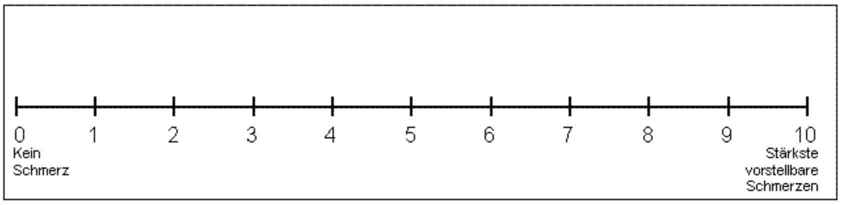

Diese Fragen betreffen Auffälligkeiten nach dem Eingriff:

3.6 Verspürten Sie direkt nach dem operativen Eingriff oder am Folgetag Übelkeit? **Ja** ○ **Nein** ○

3.7 Mussten Sie sich direkt nach dem operativen Eingriff oder am Folgetag brechen? **Ja** ○ **Nein** ○

3.7.1 Wenn ja, wie oft? **1-2 Mal** ○ **Häufiger** ○

3.8 Verspürten Sie ab dem 1. Tag nach dem Eingriff Missempfindungen, wie Kribbeln, Taubheitsgefühl oder Lähmungen an dem operierten Bein/Arm? **Ja** ○ **Nein** ○

3.8.1 Wenn ja, wie lange haben diese Missempfindungen angehalten? ___________________

3.8.2 Wenn ja, welche Missempfindungen haben Sie wahrgenommen?____________________

3.9 Haben Sie im Aufwachraum das Gefühl gehabt, nicht „Wasser lassen“ / urinieren zu können?

Ja ○ **Nein** ○

3.9.1 Wenn ja, wie lange hat dieses Gefühl noch angehalten?__________________________

3.10 Haben Sie während der Intervention Träume gehabt?

Ja ○ **Nein** ○

3.10.1 Wenn ja, waren diese unangenehm?

Ja ○ **Nein** ○

____

4. Zufriedenheit

4.1 Waren Sie mit dem Verfahren Ihrer Schmerzausschaltung während des Eingriffes zufrieden?

(Benotung nach Schulnoten)

Schulnote (1 - 6): __

4.2 Würden Sie einem Bekannten dieses Verfahren der Schmerzausschaltung weiterempfehlen? **Ja** ○ **Nein** ○

Vielen Dank für Ihre Mithilfe, Ihr verantwortlicher Anästhesist,
